# IL-12 Family Cytokines in Cancer and Immunotherapy

**DOI:** 10.3390/cancers13020167

**Published:** 2021-01-06

**Authors:** Bhalchandra Mirlekar, Yuliya Pylayeva-Gupta

**Affiliations:** 1 Lineberger Comprehensive Cancer Center, The University of North Carolina at Chapel Hill School of Medicine, Chapel Hill, NC 27599, USA; rmirlekar@med.unc.edu; 2 Department of Genetics, The University of North Carolina at Chapel Hill School of Medicine, Chapel Hill, NC 27599, USA

**Keywords:** IL-12 family cytokines, tumor microenvironment, cancer immunotherapy, anti-tumor immunity, STAT, B cell, T cell

## Abstract

**Simple Summary:**

The IL-12 family cytokines play an important role in regulating the tumor immune contexture. Recent efforts geared towards the development of better immune therapeutic approaches have identified the need to overcome immune suppression and improve the quantity and quality of anti-tumor effector immune cells within the tumor milieu. In this review, we summarize the recent findings on IL-12 family cytokines in regulating anti-tumor immunity as well as the effectiveness and benefits of enhancing anti-tumor immunity in pre-clinical and clinical settings by targeting IL-12 family cytokines.

**Abstract:**

The IL-12 family cytokines are a group of unique heterodimeric cytokines that include IL-12, IL-23, IL-27, IL-35 and, most recently, IL-39. Recent studies have solidified the importance of IL-12 cytokines in shaping innate and adaptive immune responses in cancer and identified multipronged roles for distinct IL-12 family members, ranging from effector to regulatory immune functions. These cytokines could serve as promising candidates for the development of immunomodulatory therapeutic approaches. Overall, IL-12 can be considered an effector cytokine and has been found to engage anti-tumor immunity by activating the effector Th1 response, which is required for the activation of cytotoxic T and NK cells and tumor clearance. IL-23 and IL-27 play dual roles in tumor immunity, as they can both activate effector immune responses and promote tumor growth by favoring immune suppression. IL-35 is a potent regulatory cytokine and plays a largely pro-tumorigenic role by inhibiting effector T cells. In this review, we summarize the recent findings on IL-12 family cytokines in the control of tumor growth with an emphasis primarily on immune regulation. We underscore the clinical implications for the use of these cytokines either in the setting of monotherapy or in combination with other conventional therapies for the more effective treatment of malignancies.

## 1. Introduction. IL-12 Family Cytokines: Composition, Signaling and Mechanism of Action

The IL-12 family cytokines are known to play essential roles in regulating innate and adaptive immune responses [1]. The functions of the IL-12 family cytokines have been widely studied in the settings of infection and auto-inflammatory diseases. The ability of these cytokines to modulate immune responses in cancer has been of significant interest. IL-12 family cytokines are typically secreted by innate immune cells but can also be secreted by adaptive immune cells depending on the disease and immune contexture. The members of this cytokine family are well known for shaping adaptive immune responses [2,3]. Due to their broad-spectrum roles in regulating immune responses, the IL-12 family cytokines are recognized as promising candidates for the modulation of anti-tumor immunity.

### 1.1. IL-12

**Figure 1:**
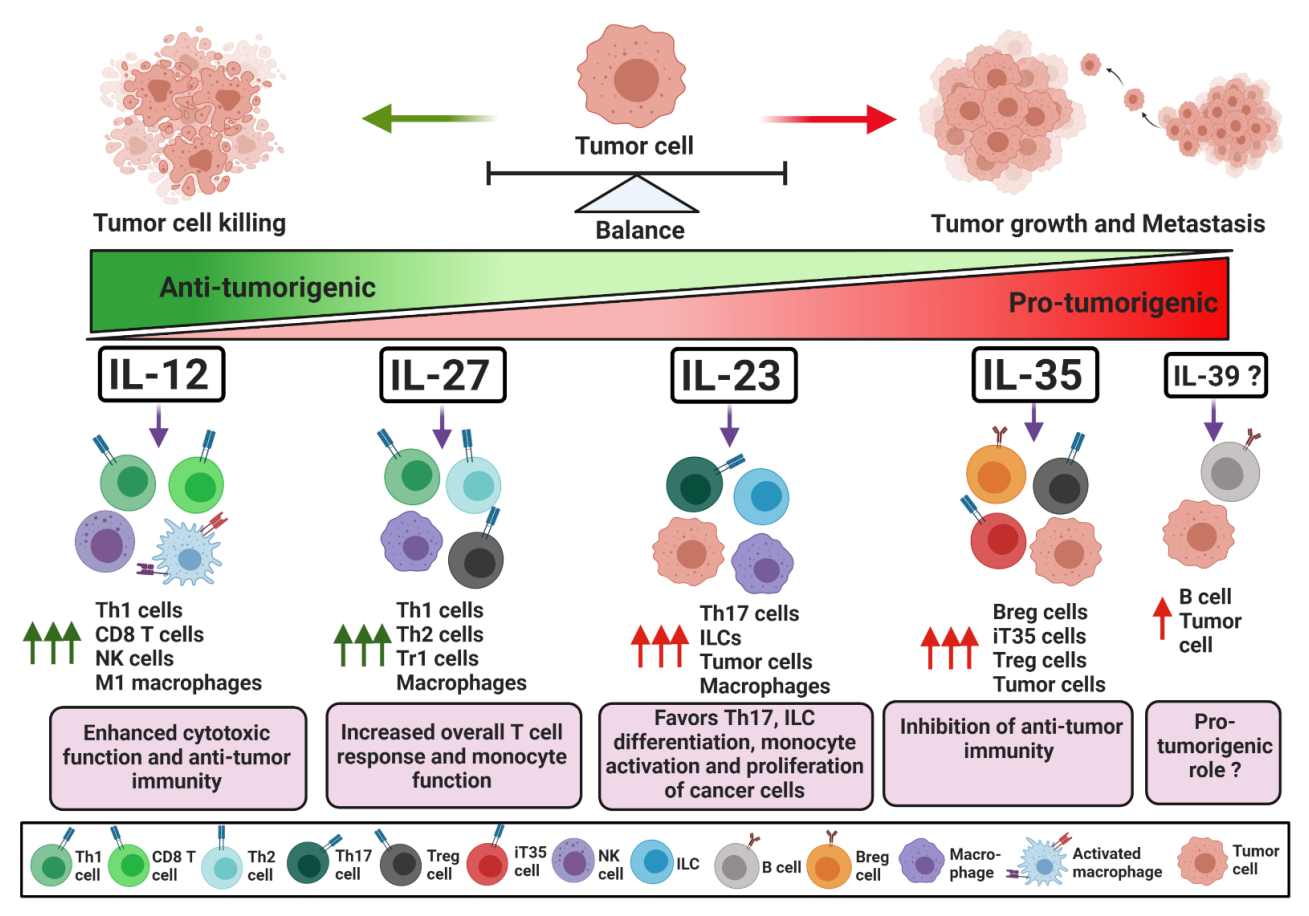
Role of IL-12 family cytokines in maintaining a balance between effector and regulatory immune responses in tumorigenesis. IL-12 activates an effector immune response against tumor cells by promoting both M1 macrophage polarization and IFN-γ- production by Th1 cells, which in turn, stimulate anti-tumor cytotoxic CD8^+^ and NK cells. IL-27 and IL-23 have dual effects on immune cells in cancer. IL-27 and IL-23 can induce an overall T cell-mediated immune response and also modulate immune suppressive macrophages. Furthermore, IL-23 can stimulate the proliferation and growth of tumor cells. Conversely, IL-35 is a strong immune suppressive cytokine; it induces regulatory B and T cell activation and proliferation that subverts anti-tumor immunity and stimulates tumor growth and metastasis. IL-39 was recently shown to be secreted by B cells and may increase cancer cell proliferation. ↑ Arrow indicates increase in respective cell type activity; Tr1—T regulatory type 1; ILC—innate lymphoid cell; Breg—regulatory B cell; Treg—regulatory T cell; iT35—IL35-inducible regulatory T cell.

IL-12 is a heterodimeric cytokine composed of p40 and p35 subunits and is considered a largely pro-inflammatory cytokine (Figure 1). It is produced by antigen-presenting cells, such as dendritic cells and macrophages, and is crucial for the recruitment and effector functions of CD8^+^ T and NK cells [4]. Therefore, IL-12 is a major contributor to effective anti-tumor immune responses [5]. IL-12 signals through IL-12Rβ1 and IL-12Rβ2 receptors expressed on target cells, which allow downstream Jak2 and Tyk2 to promote the phosphorylation of and homo-dimerization of STAT4. The homodimer of pSTAT4 binds to its target genes and regulates gene expression [1]. In CD4^+^ T cells, STAT4 activation by IL-12 is required for the transcription of T-bet, a positive regulator of Th1 cell differentiation. T-bet enhances the expression of Th1-specific cytokines, chemokines, and Th1’s associated receptors. T-bet alone can positively regulate the expression of IFN-γ, while in combination with STAT4, it enhances transcription of CXCR3, IL-12Rβ1, CCL3 and CCL4 [6,7,8]. CCL3 and CCL4 are required for the intra-tumoral recruitment of cytotoxic NK cells and CD8^+^ T cells [9,10,11]. In the presence of IL-12, NK cells are activated, express CD69 and CD25, and can further proliferate in the tumor niche [12,13]. Activated Th1 and NK cells proliferate and infiltrate into the tumor, where Th1 cells support the effector functions of tumor-specific cytotoxic T cells [4,12,13]. The IFN-γ, granzyme, and perforin secreted by cytotoxic NK and CD8^+^ T cells can induce the apoptosis of cancer cells and control tumor growth. Moreover, IL-12 facilitates antigen presentation by upregulating MHCI on tumor cells, favoring polarization to M1 macrophages and attracting effector immune cells by enhancing the production of the chemokines CXCL9, CXCL10 and CXCL11 [5,14,15,16]. Additionally, T-bet and STAT4 act as negative regulators for RORγt and Foxp3, transcription factors responsible for Th17 and Treg generation, respectively, and limit their proliferation in the tumor microenvironment (TME) [17,18,19,20]. IL-12 can also neutralize signaling by negative regulatory receptors on CD8^+^ T cells. For example, IL-12 downregulates PD-1 and IFNγR2 expression on CD8^+^ T cells, protecting tumor infiltrating CD8^+^ T cells from IFN-γ-induced cell death [21]. The activation of anti-tumor immunity by anti-PD1 requires IL-12-mediated crosstalk between T cells and dendritic cells that enables CD8^+^ T cell-mediated tumor cell killing [22].

Besides its function in effector immune cells, IL-12 alters the plasticity of terminally differentiated Treg cells by converting Foxp3^+^ Treg cells to IFN-γ-producing Foxp3^+^ T cells. Treatment with IL-12 diminishes the level of IL-2, which is required for Treg cell survival and expansion [23]. IL-12 was shown to stimulate the IFN-γ-mediated inhibition of mouse Treg cell expansion. Mechanistically, IL-12-induced IFN-γ signaling causes cell cycle arrest in Treg cells and inhibits tumor-induced Treg cell proliferation. [23,24]. These studies demonstrate that IL-12 is not only required for the activation of effector anti-tumor immune responses but can also directly inhibit immune suppression. Thus, the use of IL-12 as a cancer immunotherapy could be beneficial in controlling tumor growth by activating anti-tumor cytotoxic immune responses. Overall, IL-12 targets and modulates T cells, NK cells and antigen-presenting cells (APCs) that regulate the fate of the anti-tumor immune response against the cancer cells.

### 1.2. IL-12 in Cancer Immunotherapy

Cytokine-based immunotherapy can be effective in the treatment of numerous malignancies. IL-12 can be considered a strong candidate for immunotherapy-based interventions, as it potentiates tumor-specific cytotoxic NK and CD8^+^ T cells that are largely responsible for tumor cell killing. However, the systemic administration of IL-12 is quite toxic; therefore, alternative methods of IL-12 delivery and/or the activation of T cells by IL-12 are needed [25,26]. To that extent, a recent report by Nguyen et al. provided comprehensive updates on the development and application of a localized delivery strategy for IL-12-based immunotherapy [27]. Wang et al. reported that systemic delivery via an oncolytic adenovirus encoding IL-12 lacking the signaling peptide reduced the toxic side effects and enhanced survival in mouse models of pancreatic cancer [26]. The nanoparticle-mediated delivery of IL-12 has also enhanced the cytotoxic activity against human hepatocellular carcinoma cells [28]. The in vitro nanoparticle-mediated delivery of IL-12 specifically into naïve CD8^+^ T cells favored their expansion and the activation of the effector phenotype [28]. Several studies have shown that IL-12 favors the survival and differentiation of naïve CD8^+^ T cells towards the effector phenotype. IL-12 acts as an anti-apoptotic factor for CD8^+^ T cells by the impediment of the activation-induced cell death of CD8^+^CD62L^hi^ naïve CD8^+^ T cells, increased T cell homing, and has shown sustainable anti-tumor activity against mouse models of melanoma [29]. These findings suggest that the priming of naïve CD8^+^ T cells with IL-12 prior to adoptive cell therapy could increase their effectiveness and anti-tumor activity. Similar preclinical studies have reported the synergistic use of IL-12 with adoptive T-cell-based immunotherapies. In a syngeneic mouse model, the administration of anti–VEGFR-2 chimeric antigen receptor (CAR) and IL-12–co-transduced T cells directly into the tumor site modified the immune suppressive environment by ablating systemic and intra-tumoral VEGFR-2^+^ myeloid-derived suppressor cells (MDSCs) [30]. Moreover, genetically engineered T cells, which expressed high levels of IL-12, were therapeutically effective against established murine B16 melanoma tumors, even in the absence of a tumor vaccine and IL-2 [31]. Although the survival of IL-12 engineered cells was low compared to endogenous T cells, they demonstrated improved functionality, were detected at a higher frequency in the melanoma, and maintained the activity of endogenous NK and CD8^+^ T cells. In clinical trials, IL-12 was shown to have anti-cancer activity against human glioma [32]. In this case, 31 high-grade glioma patients were treated with human IL-12 vector (Ad-RTS-hIL-12) in a multicenter phase 1 dose-escalation trial (NCT02026271), and showed evidence of increased IFN-γ and PD-1^+^ tumor-infiltrating lymphocytes [32]. These findings suggest that increases in the concentration of intra-tumoral IL-12 improve the efficacy of adoptive T cell therapies.

**Figure 2:**
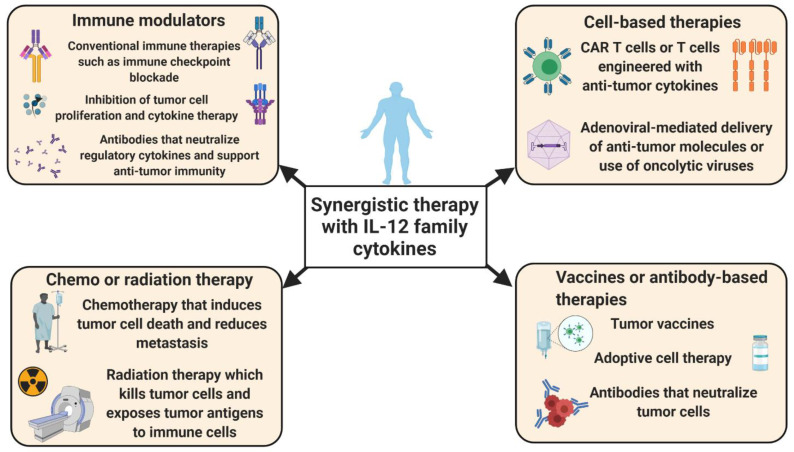
The therapeutic modulation of IL-12 family cytokines may enhance the efficacy of conventional therapy. Recent studies indicate that the therapies targeting (either upregulating or downregulating) the IL-12 family cytokines in combination with other standard therapies may increase treatment effectiveness. Context-dependent functions of IL-12 cytokines in cancer could drive the development of inhibitor or augmentative therapy axes, respectively. Drugs or antibodies targeting IL-12 family cytokines may help to restrain immune suppression within the tumor microenvironment (TME) and allow for the infiltration and proliferation of anti-tumor immune cells. Additionally, the targeted delivery of these cytokines with the help of adenovirus or chimeric antigen receptor (CAR) T cells may enhance cytotoxicity and tumor cell clearance. Such approaches could make tumor cells more sensitive to radiation, chemotherapy and immune checkpoint blockade therapy.

Another recent advance in potentiating T cell-based anti-tumor activity has been increasing the load of IL-12 in CAR T cells. In a recent pre-clinical mouse study, Kueberuwa et al. used CAR T cells expressing IL-12 and showed that modified CAR T cells were able to cure B cell lymphoma and improve the long-term survival rate [33]. In this setting, IL-12-engineered CAR T cells recruited host immune cells to elicit an anti-tumor immune response. In addition, IL-12-producing CAR T cells directed against ovarian cancer cells showed robust proliferation and secretion of IFN-γ, which resulted in increased survival in a mouse model [34]. Similar preclinical studies in a hepatocellular carcinoma model indicated that IL-12-expressing CAR T cells produced high levels of effector cytokines, accompanied by attenuated Treg cell infiltration and induced tumor cell lysis [35]. In summary, these observations reveal that the inducible expression of IL-12 improves the anti-tumor functions of CAR T cells and might provide a promising treatment strategy for cancer patients (Figure 2). Nevertheless, CAR T or TCR T cells expressing IL-12 resulted in severe, edema-like toxicity and increased serum levels of IFN-γ and TNF-α in mice with melanoma [36]. Additionally, IL-12 overexpressing CAR T cells can lead to cytokine release syndrome (CRS) and cause systemic inflammatory responses in patients treated with CAR T cells [37,38]. These severe reactions should be considered before the use of genetically engineered T cells, particularly IL-12-producing CAR T cells.

While conventional immune checkpoint blockade, such as anti-PD-L1, is commonly used for the treatment of many cancers, its efficacy is not universal, and combination therapies that augment T cell responses are needed. Hewitt et al. demonstrated that intra-tumoral IL-12 mRNA (MEDI1191) therapy was able to stimulate IL-12 production within the tumor milieu without toxic effects [39]. MEDI1191 is currently being assessed in a phase I trial in patients with solid tumors (NCT03946800). The combination of this approach with anti-PD-L1 enhanced anti-tumor immunity by promoting IFN-γ^+^ Th1 cell differentiation. Fallon et al. engineered the fusion of two molecules of murine IL-12 (NHS-muIL12) with a longer half-life than recombinant murine IL-12 [40]. The combination of NHS-muIL12 and anti-PD-L1 boosted T cell activation and effector function within the TME and augmented tumor regression in murine tumor models. Other immunotherapy combinations could also be used. For example, combined immune therapy with IL-12 and tumor necrosis factor-related apoptosis-inducing ligand (TRAIL) in humanized mouse models of hepatocellular carcinoma increased the infiltration of IFN-γ-producing NK cells and promoted the apoptosis of cancer cells. Additionally, in the presence of IL-12, TRAIL enhanced MHCI expression on antigen-presenting cells and downregulated the expression of intra-tumoral vascular endothelial growth factor (VEGF) and CD31 [41]. Another study showed that a combination of the immune-modulating protein aggregate magnesium–ammonium phospholinoleate–palmitoleate anhydride (P-MAPA) and human rIL-12 significantly reduced the migratory potential and invasion capacity by inducing the apoptosis of ovarian cancer cells [42]. These findings reveal that the use of IL-12 could substantially increase the effectiveness of cancer immunotherapy.

IL-12 may also have a beneficial role in synergizing with chemotherapy treatments. In patients with metastatic HER2^+^ cancers, a synergistic treatment of IL-12 with chemotherapy, such as trastuzumab, stimulated NK cell activity [43]. Similar synergistic approaches could be used with radiation therapy. Deplanque et al. showed that radiation-induced immune suppression in well-established tumors could be overcome by increases in IL-12-dependent Th1 responses in mouse models of colon cancer [44]. Recent reports showed that radiation therapy in combination with IL-12 induced clonal epitope-specific T cell expansion and infiltration, blocked tumor growth and the improved survival of animals with human rhabdomyosarcoma xenografts [45]. Human recombinant IL-12 was also shown to have a protective role in cancer patients treated with radiation therapy. In this setting, it diminished the complications that can arise from radiation therapy, such as severe myelosuppression or pancytopenia [46]. In murine pancreatic cancer, immunotherapy using IL-12^+^ microspheres in combination with stereotactic body radiation therapy induced intra-tumoral IFN-γ production, repolarized myeloid suppressors, promoted robust T cell activation and efficiently eliminated established liver metastases [47]. Overall, these observations reveal the importance of IL-12-based therapies in the initiation and stimulation of anti-tumor immune responses.

### 1.3. IL-23

IL-23 is a heterodimeric cytokine made up of p40 and p19 subunits. In cancer, IL-23 has been shown to have both pro- and anti-tumorigenic roles (Figure 1). Here, we discuss the dual role of IL-23 in modulating effector and/or regulatory immune responses in cancer.

#### 1.3.1. IL-23 as a Suppressor of Anti-Tumor Immunity

The pro-tumorigenic role of IL-23 was first reported by Langowski et al., where it was observed that the genetic deletion or blockade of IL-23 in mice led to an increased infiltration of cytotoxic T cells with protective effects against cancer [48]. IL-23 can be secreted by dendritic cells, monocytes, neutrophils and innate lymphoid cells (ILCs) [49,50,51,52]. Upon stimulation, macrophages, dendritic cells and neutrophils have been shown to secrete IL-23 [53]. For example, IL-6, VEGF, CCL22 and/or PGE2 produced by tumor cells recruited tumor-associated macrophages, which in turn produced IL-23 and maintained suppressive Treg activity in the TME [54,55,56,57,58]. IL-23 produced by tumor-educated neutrophils activated downstream AKT and p38 pathways in mesenchymal stem cells and transformed those mesenchymal stem cells into cancer-associated fibroblasts [59]. CXCL5 could also induce the expression of IL-23 in neutrophils to enable the enhanced migration and invasion of gastric cancer cells [60]. Microbial products could also activate intra-tumoral myeloid cells, which in turn produced IL-23 and promoted colorectal neoplasms in mice [61]. Similar studies by Jin et al. reported that microbiota stimulated myeloid cells to induce IL-23 production in mouse models of lung cancer [62].

The role of IL-23 in regulating Th17 cell differentiation is widely studied and has been shown to play a critical role in Th17 cell expansion and the maintenance of the Th17 phenotype. IL-23 is required for the stabilization of RORγt, a transcription factor for Th17 cell generation, and facilitates the secretion of the effector cytokines IL-17 and IL-21 by Th17 cells [63,64]. IL-23 also stimulates IL-17 production from γδ T cells, which can support tumor growth [62,65,66,67]. IL-23 signals through IL-12Rβ1 and IL-23R in target cells and activates Jak2 and Tyk2, resulting in the phosphorylation of STAT3 and STAT4 [1]. IL-23R signaling in CD4^+^ T cells activates STAT3, which in turn stabilizes RORγt expression and enhances IL-17 gene transcription [63,64]. IL-23 or IL-23-induced IL-17 signaling also activates NFĸB, which regulates genes responsible for the recruitment of tumor-associated macrophages (TAMs) and MDSCs in the TME [68,69,70].

Thus, IL-23 is recognized as mediator of the Th17 response and plays an important role in shaping immune responses towards cancer. For example, CCL20 produced by tumor cells cooperated with stromal IL-23 to recruit IL-17-producing Th17 cells in the TME [71,72,73,74]. IL-17, in turn, promoted tumor angiogenesis by inducing angiogenic factors, such as VEGF and PGE2, and activated oncogenic STAT3 signaling, which was essential for the expression of pro-survival and pro-angiogenic genes [72,73,74].

IL-23 can also enhance inflammation in the TME and promote tumorigenesis independently of IL-17. The elevated expression of IL-23R on tumor cells enhanced tumor-associated inflammation and promoted the development and metastasis of cancer [75,76]. As one example, IL-23 induced the activation of STAT3 in lung cancer cells expressing IL-23R and stimulated their proliferation [77]. The expression of IL-12Rβ2 and IL-23R on laryngeal tumor cells mediated crosstalk between the cancer cells and tumor-infiltrating lymphocytes and affected the prognosis of laryngeal cancer patients [78]. Furthermore, the expression of IL-23R on B-acute lymphoblastic leukemia (B-ALL) cells in B-ALL patients was increased compared to normal B-lymphocytes and acted to upregulate miR15a and downregulate the pro-survival factor BCL2 [79]. These results indicate that, apart from regulating the Th17 response, IL-23 can also potentially alter the fate and function of cancer cells.

#### 1.3.2. IL-23 as an Activator of Anti-Tumor Immunity

IL-23 is known to activate and maintain the expression of Th17-specific transcription factors, and these transcription factors can negatively regulate the immune-suppressive Treg cell response. IL-23 signaling can repress Treg differentiation and maintain the active transcription of IL-17, IL-21, IL-22 and IL-23R [63,64]. IL-23 can also regulate the function of diverse subsets of immune cells in the TME. For example, IL-23 upregulated the expression of IL-23R on type 3 innate lymphoid cells (ILC3), granulocytes and NK cells, which in turn induced their pro-inflammatory cytokine production and cytotoxic function [80,81,82]. In the presence of IL-23, intraepithelial cells secreted IL-22, which augmented their barrier function and maintained the CD4^+^ T cells’ effector phenotype [83,84,85]. On the other hand, activated fibroblasts secreted CCL5 and MCP-1, which recruited IL-23-producing macrophages [86,87]. IL-23^+^ macrophages expanded anti-tumorigenic Th17 cells that had a distinct phenotype known as Th1-like Th17 cells and promoted tumor-specific immune responses by secreting IFN-γ, CXCL9 and CXCL10 [86,87]. Thus, IL-23 and IL-17 have been shown to play multipronged roles in tumorigenesis by activating or inhibiting effector anti-tumor immunity. These findings demonstrate that IL-23 could modulate pro- as well as anti-tumor immune responses (Figure 1).

Overall, IL-23 uses different downstream signaling pathways in tumor and immune cells that regulate pro-inflammatory and anti-inflammatory pathways. Overall, context-dependent functions of IL-23 in cancer could drive the development of inhibitor or augmentative therapy axes, respectively.

### 1.4. IL-23 in Cancer Immunotherapy

IL-23 is an important player in inflammatory responses, and IL-23-producing and responding cells are highly abundant in the tumor milieu [88,89]. In fact, the abundance of IL-23 in tumors is a general feature of cancer [85,88,90]. The levels of IL-23 and IL-23R were found to be significantly higher in breast cancer tissues and positively correlated with the patient’s tumor size, TNM stage and metastasis [90]. In colorectal cancer, the levels of IL-23 in patients’ serum samples were found to gradually increase with tumor stage progression [91,92]. IL-23 could activate Th17, ILC3, granulocytes, NK cells and intra-epithelial lymphocytes, which exaggerated gut inflammation and promoted the growth of colon cancer [93]. In multiple myeloma patients, IL-23 induced IL-17 and RORC in the bone marrow microenvironment [94]. Similar studies reported that IL-23 could promote IL-17-mediated tumorigenesis by converting type 1 innate lymphoid cells (ILC1) into ILC3, resulting in poor prognosis for patients with lung carcinoma [95]. Moreover, the overexpression of IL-23 mRNA was observed in serum samples of breast cancer patients, where IL-23 played a pro-tumorigenic role by upregulating the expression of regulatory cytokines [96]. Liu et al. observed elevated levels of serum IL-23 from hepatocellular carcinoma patients, and this was associated with poor clinical outcomes [97]. They demonstrated that ILC3 cells were the main source of IL-23 in hepatocellular carcinoma, and these cells directly suppressed the CD8^+^ T cell response by enhancing apoptosis and preventing the proliferation of CD8^+^ T cells [97].

Prostate cancer patients can exhibit resistance to androgen-deprivation therapy, a condition known as castration-resistant prostate cancer (CRPC). Increased levels of IL-23 were also observed in blood and tumor samples of patients with CRPC [98]. The major source of IL-23 was shown to be MDSCs. In a mouse model of CRPC, the blockade of IL-23 improved sensitivity to androgen deprivation and synergized with conventional therapies to boost anti-tumor immunity [98]. Another study showed that elevated IL-23 was associated with poor survival in renal cell carcinoma patients [99]. Mechanistically, intra-tumoral IL-23^+^ macrophages augmented both Treg cell proliferation and IL-10/TGF-β secretion and suppressed the cytotoxic functionality of CD8^+^ T cells. The blockade of IL-23 promoted CD8^+^ T cell cytotoxicity and improved the overall survival of mice. Moreover, the blockade of IL-23 together with immune checkpoint blockade augmented therapeutic benefits [99]. Therefore, blockade of IL-23 in combination with immune checkpoint inhibitors can be a potential therapeutic approach in several cancer types (Figure 2). Conversely, reports from pancreatic cancer patients found that the levels of IL-23 decreased with tumor progression, and long-term survivors had increased IL-23 expression [100,101]. Similarly, IL-23 was shown to play a dual role in premalignant oral lesions to cancer in mice, where IL-23 was pro-inflammatory in premalignant oral lesions and inhibitory as the lesions progressed to cancer [102]. Thus, IL-23 may have distinct effects on anti-tumor immunity [103,104].

Sheng et al. showed that IL-23 can also enhance metastasis through the upregulation of anti-apoptotic factors in cancer cells [90]. In this case, the neutralization of IL-23p19 reduced cell proliferation and induced cell apoptosis by reducing the expression of BCL2 in breast cancer cell lines [90]. Besides its effects on the survival of tumor cells, IL-23 induced the expression of the proliferative marker Ki67 and endothelial marker CD31, promoting tumor cell proliferation, mammary tumor growth and pulmonary metastasis in mouse models of breast cancer [105]. Apart from the role of IL-23 in modulating the tumor cell cycle and growth, IL-23 exerts its effects on immune cells within the TME. IL-23 signaling stimulates the expression of regulatory genes, such as *IL-10*, *TGF-β* and *VEGF*, and increases the infiltration of immunosuppressive M2 macrophages and neutrophils that reduce the ability of effector CD4^+^ and CD8^+^ T cells to infiltrate tumors [105,106]. Zhang et al. showed that IL-23 is involved in the formation of immune-tolerant and pro-angiogenic TME through the induction of IL-17; this promoted the development of invasive prostate adenocarcinomas in mice [107]. These studies revealed the effects of IL-23 on the modulation of both immune cells and cancer cells. Thus, neutralizing IL-23 may augment tumor-specific immune responses and may control tumor cell proliferation and metastasis in certain cancer types.

Some preclinical studies have shown that IL-23 depletion combined with CAR T cells promotes tumor suppression, and neutralizing IL-23 can be effective particularly in combination with CAR T cell therapy [108]. The monoclonal antibody (mAB)-mediated depletion of IL-23 in mouse models of prostate cancer reinstated sensitivity to androgen deprivation therapy [98]. In one preclinical study, depleting IL-23mAB therapy in combination with CAR T cell therapy showed promising effects in the treatment of prostate cancer. The CAR T cells were generated by dual targeting using an IL-23-specific antibody and prostate-specific membrane antigen (PSMA)-specific mAB. IL-23mAB/PSMA CAR T cells had greater effectiveness than PSMA CAR only in the suppression of prostate cancer growth. Mechanistically, treatment with IL-23mAB/PSMA-CAR significantly enhanced CD45RO^+^CD8^+^ and CD127^+^CD4^+^ CAR T cells and indicated a potential role for IL-23 in the efficacy of CAR T cell therapy against prostate cancer [108,109]. By contrast, IL-23-expressing CARs were also shown to have anti-tumorigenic properties in preclinical studies. Ma et al. engineered the expression of the p40 subunit of IL-23 in T cells (p40-Td cells), and this enhanced the proliferation of activated T cells via autocrine signaling [110]. They also demonstrated in xenograft and syngeneic mouse models that p40-Td CAR T cells had enhanced anti-tumor capacity, with increases in granzyme B and decreases in PD-1 expression. Using neuroblastoma and pancreatic cancer models, they observed that p40-Td CAR T cells had greater efficacy in comparison to unmodified CAR T cells and exhibited diminished side effects in comparison to IL-18- or IL-15-producing CAR T cells [110]. Thus, the immune therapeutic strategies that target IL-23 may have use in the treatment of cancer.

### 1.5. IL-27

IL-27 is a heterodimeric cytokine consisting of EBi3 and p28 subunits [1]. IL-27 is generally involved in the differentiation and activation of different CD4^+^ T cell subsets (Figure 1). Antigen-presenting cells stimulated by tumor antigens and CD40 can secrete IL-27, which has a dual function in regulating immune responses against cancer [111].

#### 1.5.1. IL-27 as a Suppressor of Anti-Tumor Immunity

IL-27 has been shown to have anti-inflammatory properties. For example, IL-27 activated STAT3 and stabilized Foxp3 expression and IL-10 transcription in T cells, leading to sustained immune suppression [112]. Such IL-10-producing T cells are known as Tr1 cells [112,113]. Tr1 cells are immunosuppressive and downregulate the cytotoxic activity of CD8^+^ T cells and NK cells, as well as preventing the production of TNF-α and IL-6 by monocytes, which are important for anti-tumor immunity [113,114]. Mechanistically, IL-27 signaling through STAT1 and STAT3 could activate the transcription factor c-Maf and induce the generation of Tr1 cells and IL-10 production by T cells [115,116,117,118]. IL-27 has also been shown to act as an anti-inflammatory cytokine by inhibiting Th2 and Th17 cell-specific immune responses and promoting the activation and proliferation of Treg cells [112,119,120]. Additionally, the activation of STAT1 and STAT3 by IL-27 can induce the expression of IL-1β, TNF-α and IL-18, which are important for the polarization of the immunosuppressive M2 phenotype [119,120]. In advanced non-small cell lung cancer (NSCLC) patients, IL-27 has also been shown to play a pro-tumorigenic role, where IL-27 induced tolerogenic dendritic cells that helped cancer cells to escape from immune surveillance [121].

#### 1.5.2. IL-27 as an Activator of Anti-Tumor Immunity

On the other hand, IL-27 produced by dendritic cells, monocytes and macrophages following TLR activation may positively regulate Th1 differentiation through STAT1 dimerization. This subsequently leads to T-bet activation and Th1-specific gene expression [122,123,124]. IL-27 can also enhance the surface expression of MHCI and MHCII in monocytes as well as co-stimulatory CD80, CD86, and adhesion molecule CD54, overall promoting the pro-inflammatory activity of T cells [125]. IL-27 signals through IL-27R (WSX-1) and gp130 receptors on target cells [1]. IL-27R and gp130 receptors recruit Jak1 and Jak2, which in turn activate STAT1 and STAT3 [1]. IL-27 plays a critical role in the development of Th1 and IL-10-producing T cells. Th1 cells express T-bet and the effector cytokine IFN-γ through STAT1 dimerization induced by downstream IL-27 signaling [124,126]. In certain scenarios, the IL-27 receptor can also activate STAT4 and STAT5. For example, IL-27 signals through STAT1 and STAT4 to induce T-bet expression in CD4^+^ T cells, which results in the upregulation of intracellular IFN-γ [124]. At the same time, the activation of STAT5 enhances the proliferation of Th1 cells in the TME [122,127]. For example, signaling through WSX-1 and gp130 on CD8^+^ T cells activated STAT1 and STAT4 and resulted in the increased expression of granzyme, perforin and IFN-γ in a T-bet-dependent manner [128,129]. In NK cells, the activation of STAT1/STAT4 by IL-27 stimulated T-bet-dependent IFN-γ production and IL-27R expression [130,131]. These overall pro-inflammatory effects of IL-27 can stimulate anti-tumor immunity and contribute to tumor cell clearance [130,131,132]. Additionally, IL-27 may antagonize the production of IL-2, which is required for T cell activation, and regulated the intensity and duration of the effector CD4^+^ T cell response [133,134].

IL-27 signaling in innate and adaptive immune cells as well as in cancer cells regulates the expression of distinct key molecules important for the activation of immune responses in the tumor milieu. To this end, IL-27 was reported to enhance the expression of DNAM-1, NKG2D and CD69 on NK cells [131]. NK cells also secreted high amounts of perforin and granzyme B and showed enhanced cytotoxic activity against cancer cells [131]. Moreover, the synergistic action of IL-27 with IL-18 activated and increased NK cell proliferation, cytotoxicity and IFN-γ production [131]. Another important aspect of IL-27 in regulating the NK cell response against uterine endometrial cancer cell (UECC) was recently reported by Zhou et al. In this study, the production of IL-27 by uterine endometrial cancer cells in the presence of rapamycin upregulated the expression of WSX-1 and gp130 on NK cells and increased their cytotoxic activity against cancer cells in a xenograft mouse model [135]. IL-27 also reduced the proliferation of human ovarian cancer cells by activating STAT3 and inhibiting Akt signaling pathways [136]. In mouse models of colon carcinoma, IL-27 induced NK cell-mediated cytotoxicity and anti-tumor immunity by activating STAT3 and triggering the expression of perforins [137].

IL-27 can also potentiate anti-tumor immunity by supporting the differentiation and expansion of myeloid progenitor cells into anti-tumorigenic M1 macrophages. For example, IL-27 was shown to act on Sca-1^+^c-Kit^+^ cells in bone marrow and promoted their differentiation into M1 macrophages in preclinical mouse studies of B16F10 melanoma and MC38 colon adenocarcinoma [138]. IL-27 also enhanced the expression of inducible nitric oxide synthase, which sustained the anti-tumor activity of macrophages within the TME [138]. Similar studies in pancreatic cancer using co-cultures of human pancreatic cell lines and TAMs reported the role of IL-27 in restricting the differentiation of TAMs into immune suppressive M2 macrophages, which enhanced the effectiveness of gemcitabine [139]. Besides the role of IL-27 in regulating the differentiation of macrophages, IL-27 can also regulate several key surface molecules on cancer cells. Carbotti et al. showed that IL-27 triggered STAT1/STAT3 phosphorylation and upregulated the expression of surface MHC class I antigen on human small cell lung cancer cells, boosting the CD8^+^ T cell response [140]. In non-small cell lung cancer patients, IL-27 promoted the anti-tumor function of myeloid cells and suppressed epithelial-to-mesenchymal transition (EMT) in cancer cells [141]. In human prostate cancer cells, IL-27 upregulated TLR3 expression and enhanced TLR-mediated cell death [142]. A relationship between IL-27 genetic polymorphisms and cancer risk has been identified. The IL-27 2905T/G genotype is linked with a decreased susceptibility to and development of cervical cancer in patients [143]. IL-27 also hindered NSCLC and squamous cell carcinoma (SCC) growth in cooperation with granulocyte and macrophage-driven necrosis, CXCL3 production, and diminished EMT-related gene expression [141]. These results indicate that IL-27 has great potential and can be utilized in the development of novel immune-therapeutic approaches.

### 1.6. IL-27 in Cancer Immunotherapy

Given the ability of IL-27 to regulate both pro-tumorigenic and anti-tumorigenic responses, the choice of the augmentation or blockade of IL-27 should be carefully evaluated given the tumor type and the immune contexture. Recent efforts have made therapeutic use of recombinant IL-27, either alone or in combination with other therapies. Majumder et al. demonstrated that treatment with rIL-27 ameliorated angiogenesis and immunosuppression in mouse models of benzo(a)pyrene (BaP)-induced lung carcinogenesis [144]. Zhu et al. also used an IL-27-expressing recombinant adeno-associated virus (AAV-IL-27) for the treatment of mouse models of lung cancer [145]. Such treatment significantly reduced tumor growth and enhanced the T cell response by downregulating the Treg cell frequency in the peripheral blood and in the TME [145]. These approaches could supplement current methods of Treg depletion, as attempts for total Treg cell depletion in cancer can result in autoimmunity. Furthermore, AAV-IL-27 drastically increased the efficacy of immune checkpoint blockade and Granulocyte Macrophage Colony Stimulating Factor (GM-CSF) vaccine treatment with no significant adverse effects [145]. Similar studies showed the recombinant adeno-associated virus (rAAV)-mediated delivery of IL-27 reduced Treg frequency and increased the effectiveness of immunotherapeutic agents in different mouse tumor models [146]. In this case, the intra-tumoral injection of AAV-IL-27 robustly increased anti-tumor immunity against plasmacytoma J558 and B16F10 mouse tumors by inducing the expression of CXCR3 in T cells and increasing responsiveness to anti-PD-1 or T cell adoptive transfer therapy [146]. These observations indicate that the intra-tumoral administration of IL-27 in combination with immune checkpoint blockade or adoptive T cell therapy could be a suitable approach for cancer treatment.

With regards to tackling the potential pro-tumor activity of IL-27, several studies have shown that IL-27 can regulate the immunosuppression and exhaustion of T cells. For example, IL-27 could also induce indoleamine 2,3-dioxygenase (IDO) and PD-L1 expression on monocytes and tumor-associated macrophages and on human prostate, breast and lung cancer cells [147,148,149,150]. Moreover, IL-27 signaling in human ovarian cancer cells activated STAT1/STAT3 and induced the constitutive expression of IDO [149]. These alterations on monocytes and tumor cells potentiate escape from immune attack. Similar studies in human lymphoma cell lines showed that the enhancement of IL-27 expression by tumor cells triggered the surface expression of PD-L1 on tumor-associated macrophages [151]. Moreover, IL-27 signaling on tumor cells activated STAT1; upregulated the expression of PD-L1 and production of IL-10, TGF-β and IDO; and downregulated MHCI expression [111,152]. Finally, IL-27 signaling in NK and CD8^+^ T cells upregulated the cell surface expression of negative regulators, such as LAG3 and Tim3, contributing to the exhaustion of the effector phenotype [153,154]. In mouse models of melanoma, the inhibitory receptor Tim-3 was a crucial regulator of T cell dysfunction in tumorigenesis. IL-27 was shown to induce the expression of nuclear factor interleukin 3 regulated (NFIL3), which in association with T-bet, induced the expression of Tim-3 and the immunosuppressive cytokine IL-10 [153]. In this case, the blockade of IL-27 signaling resulted in reduced NFIL3 and Tim-3 expression, and decreased T cell exhaustion [153]. Similar studies reported pro-tumorigenic action of IL-27 favoring the expansion of Treg cells, where IL-27R^−/−^ Treg cells were unable to suppress anti-tumor immunity in mouse models of melanoma [155]. Furthermore, IL-27 secreted by tumor-infiltrated neutrophils upregulated CD39 expression and enhanced the immune suppressive capacity of CD163^+^ macrophages [156]. Here, the depletion of IL-27 attenuated the expression of PD-L1 and IL-10 in macrophages isolated from ovarian cancer patients [156]. These studies indicate that the IL-27/STAT3 axis can be a potential target for immunotherapy.

### 1.7. IL-35

IL-35 is a recent addition to the IL-12 family of cytokines and is composed of the p35 and EBi3 subunits [1,3]. IL-35 is a potent immune suppressive cytokine produced mostly by regulatory B (Breg) cells, iT35 cells and Treg cells and can play an important role in the suppression of effector immune responses [157]. IL-35 has also recently been detected in macrophages, dendritic cells and tumor cells [158,159,160]. The role of IL-35 in tumor growth is becoming more prominent due to its ability to inhibit effector immune responses (Figure 1). IL-35 regulates the differentiation of CD4^+^ T cells and strongly favors regulatory T cell fate [161]. IL-35 is also thought to regulate the Th1 and Th17 lineage-specific transcription factors T-bet and RORγt as well as cytokines IFN-γ and IL-17 [162,163,164]. For example, IL-35 suppressed the effector functions of CD4^+^ cells and favored tumor growth by facilitating the exhaustion of CD8^+^ T cells [165,166]. Additionally, IL-35 restricted CD8^+^ T cell activation by suppressing the expression of the costimulatory surface molecule CD28, and Th1 cytokine production, and reduced cytolytic functions by repressing the expression of perforins [167,168]. Furthermore, IL-35 converted effector T cells into IL-35-producing T cells known as iT35 cells and activated IL-35 production and the proliferation of Treg cells [163,169,170]. A subset of mouse and human regulatory B cells can be a major source of IL-35 in certain malignant tumor types [167,171,172]. Additionally, IL-35 can enhance its own expression in B cells through IL-12Rβ2 and IL-27Rα and the activation of STAT1 and STAT3, resulting in the augmented formation of Breg cells [173,174]. Mechanistically, the loss of IL-35 hindered pancreatic tumor growth via increases in effector CD4^+^ T cells and CD8^+^ T cells within the TME [165]. A specialized subset of B cells marked by CD21^hi^CD1d^hi^CD5^+^ was identified as the main source of IL-35 in pancreatic cancer. The B cell-specific production of IL-35 promoted the expansion of Treg cells and blocked the anti-tumor activity of effector CD4^+^ T cells [169]. Furthermore, IL-35 directly acted on CD8^+^ T cells and suppressed their infiltration and effector function by downregulating the expression of IFN-γ and CXCR3 in a STAT3-dependent manner [169]. IL-35-producing B cells were also elevated in the peripheral blood of gastric cancer (GC) patients and were responsible for the accumulation of immune suppressive Treg cells, MDSCs, IL-10-producing B cells and CD14^+^ monocytes [170].

IL-35 signals through IL-12Rβ2 and gp130 receptors in T cells, which triggers Jak1 and Jak2 to activate STAT1 and STAT4 [157]. IL-35-mediated signaling via STAT1/STAT4 in conventional T cells can polarize them to IL-35-producing iT35 cells [171]. The expression of IL-35 by Foxp3^+^ Treg cells can reduce the proliferation of effector T cells and can lead to T cell exhaustion [171,172]. In this regard, Turnis at al. also demonstrated that IL-35^+^ Treg cells could enhance the surface expression of the negative regulators Tim-1, PD-1 and LAG3 on anti-tumor T cells [166]. A clinical study also showed that IL-35 produced by Treg cells promoted the growth of acute myeloid leukemia (AML) blasts in adult AML patients by limiting the activity of CD4^+^CD25^−^ T effector cells and increasing cancer cell proliferation [175]. IL-35 was reported to limit anti-tumor immunity in NSCLC patients by suppressing Th1 and Th17 responses and cytotoxic genes in CD8^+^ T cells [176]. Apart from T cells, IL-35^+^ macrophages were able to upregulate IL-12Rβ2 expression on cancer cells and make them more responsive to IL-35, facilitating the activation of JAK2–STAT6 and GATA3, and increased metastasis in mouse models of breast and lung cancer [159]. Moreover, Zou et al. showed that IL-35 facilitated the polarization of neutrophils, leading to increases in tumor growth in mouse models of melanoma and hepatocellular carcinoma [177]. Furthermore, IL-35 promoted immune suppression by favoring M2 macrophage polarization [178,179]. IL-35 also impeded DC maturation by reducing the cell surface expression of MHCI and MHCII and co-stimulatory molecules CD80/CD86 [180,181,182]. Overall, these observations indicate that IL-35 has a strong immune suppressive effect in the TME and could play a major role in controlling anti-tumor immunity; therefore, targeting IL-35 in cancer may hold substantial therapeutic potential for the treatment of patients with cancer.

### 1.8. IL-35 in Cancer Immunotherapy

A detailed understanding of IL-35 in cancer is essential. This information may lead to the development of new immunotherapeutic agents for the treatment of cancer patients, and it can be used as a potential biomarker for disease progression. As an example, elevated levels of IL-35 have been associated with poor prognosis in many solid cancer types [183,184,185,186].

#### 1.8.1. IL-35 in Inhibiting Tumor Growth

Although IL-35 plays a pro-tumorigenic role in several cancer models, such as melanoma, pancreatic cancer, NSCLCC, breast cancer, lymphoma and gastric cancer, among others, Zhang et al. reported that IL-35 inhibited the cell migration, proliferation, colony formation and invasion of human colon cancer cells by suppressing β-catenin [187]. In this instance, IL-35 suppressed cancer growth in vivo and sensitized colon cancer cells to chemotherapy. Thus, IL-35 in combination with chemotherapy could be a useful treatment for colon cancer. Similar studies showed that IL-35 plays an inhibitory role in human NSCLC and colon cancer cells by suppressing cell migration and colony formation [188,189]. In hepatocellular carcinoma (HCC) patients, IL-35 expression was significantly reduced in patients with advanced cancer as compared to early stages. The HCC patients with reduced IL-35 had increased tumor size, distant metastases and positive microvascular invasion. Furthermore, the overexpression of IL-35 upregulated MHCI and CD95 in HepG2 cancer cells and reduced cell migration, colony formation and invasion. These data indicate that IL-35 plays an anti-tumorigenic role in HCC by enhancing MHCI-specific anti-tumor immunity and by reducing cancer cell migration. [190].

#### 1.8.2. IL-35 in Promoting Tumor Growth

However, most research studies have reported that increased levels of IL-35 can be correlated with the pathogenicity and progression of cancer. The levels of IL-35 in the serum samples of prostate cancer patients were highly elevated compared with healthy controls and correlated with disease progression [184,191]. Similar studies reported elevated levels of circulating IL-35 in breast cancer patients, which positively correlated with the expression of Ki67, p53 and EGFR on breast cancer cells [192]. Furthermore, high levels of IL-35 were shown to be associated with the poor survival of diffuse-large B-cell lymphoma (DLBCL) patients treated with chemotherapy [193]. Additionally, increased levels of IL-35 were observed in the bone marrow of acute myeloid leukemia (AML) patients and correlated positively with clinical stages [194]. IL-35 can also be secreted by cancer cells and was shown to have a pro-tumorigenic effect particularly in pancreatic cancer. To this end, IL-35 was associated with poor prognosis in pancreatic cancer patients and is also thought to promote the growth of pancreatic cancer cells by enabling metastasis and extravasation [195]. In this case, IL-35 signaling in cancer cells activated STAT1 via the gp130 receptor, triggered the expression of ICAM1, and increased endothelial adhesion and metastasis. The human pancreatic cancer cell production of IL-35 also favored the proliferation of tumor cells by promoting the expression of cyclin B, cyclin D, Cdk2 and Cdk4, and it inhibited apoptosis by increasing levels of Bcl2 and decreasing levels of TRAILR1 [196]. Jin et al. showed that elevated levels of IL-35 in pancreatic cancer patients correlated with increases in tumor size, TNM staging and lymph node metastasis [197]. Furthermore, IL-35 produced by human nasopharyngeal carcinoma, B cell lymphoma and melanoma tumor cells enhanced the infiltration of CD11b^+^Gr1^+^ myeloid cells and promoted angiogenesis [160]. These findings indicate that IL-35 may have a distinct role in cancer when produced by immune cells or cancer cells.

Accumulating evidence suggests that IL-35 blockade could synergize with immunotherapy to ameliorate tumor growth. Turnis et al. showed that anti-PD-1 therapy combined with the depletion of IL-35 and/or deletion of Treg cell-specific IL-35 expression significantly limited murine melanoma and colon cancer growth by promoting cytotoxic T cell proliferation, effector function and long-term memory formation [166]. Similarly, the loss of B cell-specific IL-35 expression or antibody-based IL-35 blockade synergized with anti-PD1 treatment in a CD8^+^ T cell-dependent manner to inhibit pancreatic cancer growth in preclinical models [169]. A similar approach of combining IL-35 blockade with the immune checkpoint inhibition of PD1 or PD-L1 was effective in NSCLC [185]. In this case, the neutralization of IL-35 hindered immunosuppressive macrophages and Treg cells while enhancing anti-tumor immunity. These findings identify the potential behind targeting IL-35-mediated immune suppression in the TME and highlight the importance of IL-35 as a therapeutic target in synergistic approaches with immune checkpoint therapy for the treatment of multiple cancer types.

### 1.9. IL-39

IL-39 is a newly proposed member of the IL-12 family. It is a heterodimeric cytokine consisting of the subunits p19 and EBi3 [198]. Most studies to date have only focused on cancer cell lines, and no concrete reports in animal models or human subjects are available yet. Thus, it is premature to designate IL-39 as an inflammatory or regulatory cytokine at this point in time. Wang et al. demonstrated that IL-39 could be secreted by LPS-stimulated B cells and could induce inflammation by activating STAT1/STAT3 in mouse models of lupus [198]. IL-39-producing B cells increased in mice with lupus, and the silencing of IL-39 led to reduced disease severity [199]. In this study, IL-39 induced the differentiation and expansion of CD11b^+^Gr1^+^ neutrophils in vivo. Furthermore, activated neutrophils could further potentiate the expression of B cell activation factor (BAFF) in GL7^+^ B cells, creating a positive feedback loop that results in the increased production of IL-39 [199]. IL-39 was also shown to have a pro-tumorigenic function in pancreatic cancer, where in the presence of IL-39, colonies of human pancreatic cancer cells significantly increased their proliferation rate [200]. This was accompanied by the reduced expression of *p21* and *TRAILR1*. IL-39 is thought to signal via IL-23R and gp130 in target cells and to activate downstream STAT1 and STAT3 signaling [201,202]. Floss et al. engineered shuffled IL-12 family cytokine receptors, which are responsive to IL-39. The authors found that IL-39 may use two additional receptor combinations, IL-23R/IL-12Rβ2 and gp130/IL12Rβ1, in Ba/F3 cells [203]. These findings may highlight the flexibility of receptor usage by IL-39. More work needs to be done in order to understand the potential of targeting IL-39 in cancer immunotherapy.

## 2. Conclusions and Future Perspectives

IL-12 family cytokines play a critical role in the regulation of innate and adaptive immune responses. Their functions in the modulation of immune responses are well reported in autoimmunity and infectious diseases. These cytokines also play key roles in cancer initiation and progression. Tumor growth and spread have a direct relationship with host immune responses, and it is clear that IL-12 family cytokines can regulate tumor growth (Figure 1). Therefore, targeting or modifying the immune response against the tumor by harnessing the biological functionality of IL-12 family cytokines has recently gained lots of attention. The silent feature of various tumors is that they escape the host immune attack by favoring immune suppression within the TME. Cancer cells can secrete immune suppressive cytokines and chemokines and, in conjunction with regulatory immune cells, hinder the activity and proliferation of tumor-specific cytotoxic cells. Due to the “cold” nature of many cancers, therapies such as immune checkpoint blockade, adoptive T cell therapy, tumor vaccines, conventional chemotherapy and/or radiotherapy are frequently unable to manifest effective responses. Thus, strategies aiming to boost immune infiltration and functionality would be highly beneficial.

In this review, we summarized recent studies and advancements in the IL-12 field, namely, the roles and potential for the targeting of IL-12, IL-23, IL-27, IL-35 and IL-39 in tumorigenesis. The overarching objective is to make a tumor more susceptible to immune attack and become more responsive to conventional therapies. Targeting these cytokines may alter the tumor phenotype from immunologically “cold” to immunologically “hot”. As discussed above, these cytokines are secreted not only by immune cells but also by tumor cells. Therefore, therapies focusing on IL-12 family cytokines can block the tumor cell cycle, induce apoptosis and prevent tumor cell proliferation, together with facilitating effector immune responses against cancer cells. Synergistic therapies that focus on IL-12 family cytokines and immune checkpoint blockade, such as anti-PD1, neutralizing antibodies, adoptive T cell therapy and CAR T cell therapy have shown promising effects in preclinical models. Localized IL-12 delivery and synergistic therapy consisting of IL-12 with immune checkpoint inhibitors and adoptive cell transfer is under investigation in clinical trials [27]. Hu et al. demonstrated that the combination of IL-12 and doxorubicin could enhance the infiltration of cytotoxic T cells into large solid tumors in different human xenograft models [204]. The local expression of IL-12 was achieved by injecting IL-12 DNA and conducting in vivo electroporation. This treatment hampered Treg cell infiltration and increased the effector functions of tumor-infiltrated T cells [204,205]. This strategy is under investigation in clinical trials (NCT01579318, NCT00323206, NCT01502293 and NCT02345330) and also in combination with pembrolizumab (NCT02493361 and NCT03132675) [205]. Although IL-12 is an effector cytokine and recruits a variety of effector immune cells at the tumoral site, it is possible that it can induce inflammatory effects. Therefore, future studies, particularly studies involving clinical trials, should consider the inflammatory effects associated with IL-12 family cytokines while using them as immunotherapy for cancer patients.

Future studies should place more emphasis on developing appropriate targeting reagents suitable for use in human clinical trials. More preclinical studies need to be performed, especially on IL-39. Given the role of IL-12 family cytokines in modulating the immune cell as well as cancer cell phenotype and receptor expression pattern, downstream signaling pathways need to be studied in detail, as they may dictate downstream therapeutic strategies. Preclinical studies using genetic mouse models need to include head-to-head comparisons across the distinct subunits that comprise IL-12 cytokines in order to establish which particular IL-12 family cytokine(s) play a role in a specific cancer type. Although IL-12 family cytokines seem to be promising candidates for the treatment of different malignancies, their associated side-effects and roles in regulating the local versus systemic immune system still need to be elaborated in detail.
